# Fuzzy fixed point approach to study the existence of solution for Volterra type integral equations using fuzzy Sehgal contraction

**DOI:** 10.1371/journal.pone.0303642

**Published:** 2024-06-06

**Authors:** Muhammad Zahid, Fahim Ud Din, Kamal Shah, Thabet Abdeljawad

**Affiliations:** 1 Abdus Salam School of Mathematical Sciences, Government College University, Lahore, Pakistan; 2 Department of Mathematics and Sciences, Prince Sultan University, Riyadh, Saudi Arabia; 3 Department of Mathematics, University of Malakand, Chakdara, Dir(L), KPK, Pakistan; 4 Department of Medical Research, China Medical University, Taichung, Taiwan; 5 Department of Mathematics and Applied Mathematics School of Science and Technology, Sefako Makgatho Health Sciences University, Ga-Rankuwa, South Africa; Federal University of Technology - Parana, BRAZIL

## Abstract

In this manuscript, we present a novel concept known as the fuzzy Sehgal contraction, specifically designed for self-mappings defined in the context of a fuzzy metric space. Our primary objective is to explore the existence and uniqueness of fixed points for self-mappings in fuzzy metric space. To support our conclusions, we present a detailed illustrative case that demonstrates the superiority of the convergence obtained with our suggested method to those currently recorded in the literature. Moreover, we provide graphical depictions of the convergence behavior, which makes our study more understandable and transparent. Additionally, we extend the application of our results to address the existence and uniqueness of solutions for Volterra integral equations.

## 1 Introduction

In the realm of mathematics, the study of fixed points and their applications has appeared as a versatile and foundational field. Fixed point theory, a branch of mathematics with a very vast history, explores the existence and properties of points that remain unchanged when subjected to specific transformations. It has found widespread applications across diverse disciplines, including physics, economics, computer science, and engineering providing beneficial insights into the gentle attitude of functions and mappings.

Fixed point theory, in its traditional form, addresses questions like, “Under what conditions does a function have a point that remains unchanged after applying that function?” This seemingly simple question leads to a wealth of mathematical exploration and has provided powerful tools for solving problems with real-world significance.

The concept of fixed points has a rich history dating back to the beginning of 19^*th*^ century, with significant contributions from mathematicians like Banach, Kakutani, and Brouwer. They laid the essential groundwork for fixed point theory by flourishing theorems and concepts that continue to shape mathematical research and practical applications today. Since then, fixed point theory has evolved in different disciplines, including functional analysis, topology, optimization, and dynamical systems.

The Banach Contraction Principle [1], named after the mathematician Stefan Banach, is a fundamental theorem in mathematics. It attests to the presence and uniqueness of a fixed point for particular categories of mappings within a metric space.. In essence, if a mapping slowly brings points closer to each other, it must have a single point that remains stationary called as a fixed point. This principle has wide-ranging applications, particularly in numerical methods and the study of dynamical systems, making it a vital tool in various mathematical disciplines.

In 1968 Bryant [2], introduced the generalization of the Banach’s principle. He attained some results in fixed point theory, where the self mapping might not be a contraction but its *n*^*th*^ power becomes a contraction therefore it gives a fixed point for the *n*^*th*^ power of self map, which also results in the fixed of the self mapping.

In 1969, V. M. Sehgal [3] also introduced a generalization of the Banach’s principle named as sehgal contraction. In this generalization V.M. Sehgal basically generalized the idea of Bryant and introduces one of the generalized contraction where for each point *ξ* in the space there exist a natural number *n*(*ξ*) depends upon *ξ* which is termed as the power of the self mapping for which the contraction condition satisfied and fixed point is attained resulting in the fixed point of the self map itself, which doesn’t satisfy the contraction condition. After that dozen of modifications and generalizations were introduced.

In 1970, L.F. Guseman [4] extended the idea of Sehgal by relaxing the continuity condition to attain fixed point for self maps as discussed by Sehgal.

On the other hand, as mathematics evolved to tackle increasingly complex and ambiguous problems, fuzzy fixed point theory naturally extends from the foundation of fixed point theory. Fuzzy fixed point theory accommodates imprecision and uncertainty of crucial aspects of many world situations.

In 1965 Zadeh [5] extend the set theory’s concept by relaxing the well-defined condition and introduced a new concept that named a fuzzy set theory. He gave an idea to deal with the ambiguity in the statements. He introduced a membership function that deals with the containment of the elements in the set and the membership function varies with different scenarios.

In 1975 Kramosil and Michalek pioneered the study of fuzzy metric spaces by applying fuzzy set theory, as described in their work [6]. The construction of fuzzy metric space has followed basic idea of the fuzzy set that deals with ambiguity. Schweizer and Sklar [7] introduced the concept of continuous *t*-norm for the fuzzy metric space.

In 1988 Grabiec, M. [8] was first to introduce the study of fixed point in the fuzzy metric space and give fuzzy version of Banach’s principle. He introduced the concept of contraction into the framework of fuzzy metrics, establishing notions of sequence convergence, Cauchy sequences, and completeness within the context of fuzzy metrics.

Recently, Mudasir, et al. gives some new results. In [9] they introduce a novel notion called “Kannan-graph-fuzzy contraction,” which combines ideas from Kannan mappings, fuzzy contractions, and graph mappings. This method provides a novel framework for solving nonlinear problems that arise in the real world and has many applications in engineering and technology. In [10] they investigates fixed point convergence in graphical *B*_*c*_–Kannan–contractions within graphical extended *b*–metric spaces. In [11] using an alternating distance function, they report new findings on coincidence, best proximity, and fixed points in multivalued proximal contractions within *b*-metric spaces. Furthermore, recent advancements in the field can be found in [12–14].

In this manuscript we construct the Sehgal type contraction in fuzzy complete metric space. We will establish our results for continuous mappings and also prove some results omitting continuity. We will embellish our work with a non-trivial example to endorse our results. Furthermore, a thorough numerical and graphical analysis is performed to compare the rates of convergence. Our results clearly demonstrate that our proposed approach exhibits a significantly faster convergence. To further reinforce the validity of our study results, we finally use our results to determine the existence and uniqueness of solutions for Volterra integral equations.

## 2 Preliminaries

We will start with all the details required to prove our main results, we display some previous defined result, notations and definitions. Let us start with the basic definition of metric space.

**Definition 2.1** [15] *Upon satisfying the given conditions, a function* ϱ:Ξ×Ξ→R, *with a range of non-negative real numbers, is considered a metric, such that*:

1. *ϱ*(*ξ*, *η*) ≥ 0,
2. *ϱ*(*ξ*, *η*) = 0 *iff* *ξ* = *η*,
3. *ϱ*(*ξ*, *η*) = *ϱ*(*η*, *ξ*),
4. *ϱ*(*ξ*, *ζ*) + *ϱ*(*ζ*, *η* ≥ *ϱ*(*ξ*, *η*), *for each ξ*, *η*, *ζ* ∈ Ξ.

*Here ϱ is a metric on* Ξ, *and the pair* (Ξ, *ϱ*) *defines the metric space, with* Ξ *identified as the ground set. The individual elements ξ*, *η*, *ζ* ∈ Ξ *are recognized as points within this metric space. Consequently, the notation* Ξ *encompasses the metric space* (Ξ, *ϱ*).

*The metric space* (*ϱ*, Ξ) *is known to be the complete metric space if and only if each cauchy sequence is converges in the metric space*.

Few examples are also presented from the literature [15] to explain the distance or metric.

**Example 2.2**. *We have few functions* $\varrho_{1},\varrho_{2},\varrho_{3}:R\times R\to R$ *given as*:

$$\begin{array}{ccc} \varrho_{1}\left(\xi ,\eta \right) & = & \sqrt{|\xi -\eta |,}\\ \varrho_{2}\left(\xi ,\eta \right) & = & |\xi -\eta |,\\ \varrho_{3}\left(\xi ,\eta \right) & = & |\frac{1}{\xi }-\frac{1}{\eta }|, \end{array}$$

*then* *ϱ*_1_ *and ϱ*_2_ *be metrics defined on the set of real numbers*, R. *Furthermore*, *ϱ*_3_ *is a metric defined on the real numbers excluding zero, denoted as* R\{0}.

**Definition 2.3** [1] *Let us take a metric space* (Ξ, *ϱ*), *and define a mapping ϑ*: Ξ → Ξ. *The mapping ϑ is known as contraction if for each ℏ* ∈ (0, 1), *The self mapping ϑ satisfy the following condition*:

$$\begin{array}{c} \varrho (\vartheta (\xi ),\vartheta (\eta ))\leq \hbar \;\varrho (\xi ,\eta ),\;\;\forall \;\;\xi ,\eta \in \Xi . \end{array}$$ (1)

**Definition 2.4** [3] *Let us take a metric space* (Ξ, *ϱ*), *and define a mapping ϑ*: Ξ → Ξ. *The mapping ϑ is known as Sehgal contraction if there exist ℏ* ∈ (0, 1), *then for each element ξ in* Ξ *there exist a positive integer n*(*ξ*) *such that*:

$$\begin{array}{c} \varrho (\vartheta^{n(\xi )}\left(\xi \right),\vartheta^{n(\xi )}\left(\eta \right))\leq \hbar \;\varrho \left(\xi ,\eta \right),\;\;\forall \;\;\eta \in \Xi . \end{array}$$ (2)

Bryant [3], gives the extension the Banach’s principle in the following way.

**Theorem 2.1**. *Let us take a complete metric space* (Ξ, *ϱ*), *and a self mapping ϑ*: Ξ → Ξ, *If ϑ*^*n*^ *satisfy the condition* (1) *then, the self map ϑ attain a unique fixed point*.

V. M. Sehgal [3] extended the idea of Bryant, and result is defined as below.

**Theorem 2.2**. *Let us take a complete metric space* (Ξ, *ϱ*), *and define a mapping ϑ*: Ξ → Ξ, *that is continuous and satisfies the Sahgal contraction for each ξ*_0_ *in* Ξ. *The mapping ϑ, then has a fixed point α*, *that is unique and ϑ*^*n*^(*ξ*_0_) → *α for each ξ*_0_ *in* Ξ.

Guseman, and Jr. [4] removed the continuity condition in Sehgal result and gives the following theorem.

**Theorem 2.3**. *Let* (Ξ, *ϱ*), *be a complete metric space and define a self mapping ϑ*: Ξ → Ξ. *Assuming that there is a subset of* Ξ *called* B, *where* ϑ(B) *is the subset of* B, *and the self mapping ϑ satisfy the Sahgal contraction condition* (2) *for each ξ*_0_ *in* B *and for some* $\xi_{0}\in B,cl\left\{\vartheta^{n}\left(\xi_{0}\right):n\geq 1\right\}\subset B$, *then the mapping ϑ has a fixed point α that is unique in* B *and ϑ*^*n*^(*ξ*_0_) → *α for each ξ*_0_ *in* B. *If ϑ satisfy the Sahgal contraction condition* (2) *for every ξ*_0_ *in* Ξ, *then the unique fixed point α exist in* Ξ, *and ϑ*^*n*^(*ξ*_0_) → *α for each ξ*_0_ *in* Ξ.

We start with the concept of the *t*-norm and the definition of fuzzy metric space before talking about some significant findings from fuzzy metric space.

**Definition 2.5**. [7] *Let us define a binary operation* ❃: [0, 1]^2^ → [0, 1], *if* ([0, 1], ❃) *is an abelian monoid with 1 (unity), where*

α❃β≤η❃γ,wheneverα≤ηandβ≤γ,∀α,β,γ,η∈[0,1],

*then we say that* ❃ *is a continuous t-norm*.

**Definition 2.6**. [6] *Let* Ξ *be a nonempty set, we define a fuzzy metric M*: Ξ × Ξ × [0, ∞) → [0, 1] *with the following conditions*:

$M_{1}$ ) *M*(*ξ*, *η*, 0) = 0,

$M_{2}$ ) *M*(*ξ*, *η*, *κ*) = 1, ∀ *κ* > 0, ⇔ *ξ* = *η*,

$M_{3}$ ) *M*(*ξ*, *η*, *κ*) = *M*(*η*, *ξ*, *κ*), ∀ *ξ*, *η* ∈ Ξ, *κ* > 0,

$M_{4}$ ) *M*(*ξ*, *η*, *κ*) ❃ *M*(*η*, *γ*, *μ*) ≤ *M*(*ξ*, *η*, *κ* + *μ*).

*Then the triplet* (Ξ, *M*, ❃), *is known as fuzzy metric space*.

Here are a few illustrative examples of fuzzy metrics space:

**Example 2.4**. *Let* Ξ = **R**. *Define* *α*❃*β* = *αβ*, ∀ *α*, *β* ∈ [0, 1]. *For* *ξ*, *η* ∈ Ξ *and* *κ* ≥ 0, *define*

$$\begin{array}{c} M\left(\xi ,\eta ,\kappa \right)=\{\begin{array}{cc} \frac{\kappa }{\kappa +d(\xi ,\eta )} & \;if\;\;\xi ,\eta \in \Xi ,\;\;\kappa >0\\ 0 & \;if\;\;\xi ,\eta \in \Xi ,\;\;\kappa =0. \end{array} \end{array}$$ (4)

*Then M is fuzzy metric on* R *and* (R,M,❃) *is known as fuzzy metric space*.

**Definition 2.7**. [8] *Let us take the sequence* {*ξ*_*n*_} *in a fuzzy metric space* (Ξ, *M*, ❃). *We say that the sequence* {*ξ*_*n*_} *is said to be converges to a point ξ in the fuzzy metric space* (Ξ, *M*, ❃), *if*

$$\begin{array}{c} \underset{n\to \infty }{\text{lim}}\;M\left(\xi_{n},\xi ,\kappa \right)=1,\;\;\;for\;each\;\;\;\kappa >0. \end{array}$$ (3)

**Definition 2.8**. [8] *When each Cauchy sequence within a fuzzy metric space converges, we declare it to be complete*.

Similarly, we can define the compactness on the fuzzy metric space. If every sequence in fuzzy metric has a convergent subsequence, the fuzzy metric space is said to be compact.

**Definition 2.9**. [8] *Let* (Ξ, *M*, ❃) *be a fuzzy metric space and we define a self mapping ϑ defined on* Ξ *into itself. We say that ϑ is fuzzy contraction if*

$$\begin{array}{c} M(\vartheta (\xi ),\vartheta (\eta ),\hbar \kappa )\geq M(\xi ,\eta ,\kappa ),\;\;\forall \;\;\xi ,\eta \in \Xi ,\;\;\hbar \in (0,1) \end{array}$$ (4)

**Theorem 2.5**. [8] *We assume that* (Ξ, *M*, ❃) *be a complete fuzzy metric space*,

$$\underset{\xi \to \infty }{\text{lim}}\left(\xi ,\eta ,\kappa \right)=1,\;\;\forall \;\;\xi ,\eta \in \Xi ,$$

*and define a self mapping ϑ*: Ξ → Ξ, *which satisfy the condition* (4). *Then the self mapping ϑ have a unique fixed point*.

## 3 Primary results on fuzzy Sehgal contraction

Let us start this section with the definition of Sehgal Fuzzy contraction in the fuzzy metric space.

**Definition 3.1**. *Let us consider* (Ξ, *M*, ❃), *be a fuzzy metric space and a self mapping ϑ from* Ξ *into itself. The mapping ϑ is said to be Fuzzy Sehgal contraction if for each ξ in* Ξ, *there exist a natural number n*(*ξ*), *such that* ∀ *y* ∈ Ξ *and ℏ* ∈ (0, 1)

$$\begin{array}{c} M(\vartheta^{n(\xi )}\left(\xi \right),\vartheta^{n(\xi )}\left(\eta \right),\hbar \kappa )\geq M\left(\xi ,\eta ,\kappa \right),\;\;\forall \;\;\xi ,\eta \in \Xi ,\;\;\hbar \in \left(0,1\right). \end{array}$$ (5)

Now, we are equipped with all basic tools. Now, move to our main results.

**Theorem 3.1**. *Let us take a complete fuzzy metric space* (Ξ, *M*, ❃), *such that*

$$\underset{\kappa \to \infty }{\text{lim}}M(\xi ,\eta ,\kappa )=1,\;\;\forall \;\;\xi ,\eta \in \Xi .$$

*Define a self mapping ϑ from* Ξ *into itself, which is continuous and satisfy condition* (5), *then the mapping ϑ has a unique fixed point ξ in* Ξ, *and ϑ*^*n*^(*ξ*_0_) → *ξ, for each ξ*_0_ *in* Ξ.

*Proof*. Let us take an element *ξ*_0_ from the complete fuzzy metric space Ξ, and *m*_0_ = *n*(*ξ*_0_), such that $\xi_{1}=\vartheta^{m_{0}}\left(\xi_{0}\right)$, and by the induction, we say *m*_*i*_ = *n*(*ξ*_*i*_) such that $\xi_{i+1}=\vartheta^{m_{i}}\left(\xi_{i}\right)$. Here, our aim to show that the sequence {*ξ*_*n*_} is convergent in fuzzy metric space Ξ. So, we first shoe the sequence {*ξ*_*n*_} is cauchy in Ξ.

$$\begin{array}{cc} M(\xi_{n},\xi_{n+1},\kappa ) & =M(\vartheta^{m_{n-1}}\cdot \vartheta^{m_{n}}\left(\xi_{n-1}\right),\vartheta^{m_{n-1}}\left(\xi_{n-1}\right),\kappa ),\\ & \geq M(\vartheta^{m_{n}}\left(\xi_{n-1}\right),\xi_{n-1},\frac{\kappa }{\hbar }),\\ & \vdots \\ & \geq M(\vartheta^{m_{n}}\left(\xi_{0}\right),\xi_{0},\frac{\kappa }{\hbar^{n}}). \end{array}$$

Now, take *ξ*_*n*+*p*_, *ξ*_*n*_ and we have by applying the triangular inequality in fuzzy metric space

$$\begin{array}{cc} M(\xi_{n+p},\xi_{n},\kappa ) & \geq M(\xi_{n+p},\xi_{n+p-1},\frac{\kappa }{\hbar })\;❃M(\xi_{n+p-1},\xi_{n+p-2},\frac{\kappa }{\hbar })\;❃\cdots \;❃M(\xi_{n+1},\xi_{n},\frac{\kappa }{\hbar }),\\ & \geq M(\vartheta^{m_{n}}\left(\xi_{0}\right),\xi_{0},\frac{\kappa }{p\hbar^{n+p-1}})\;❃M(\vartheta^{m_{n}}\left(\xi_{0}\right),\xi_{0},\frac{\kappa }{p\hbar^{n+p-2}})\\ & \;❃\cdots \;❃M(\vartheta^{m_{n}}\left(\xi_{0}\right),\xi_{0},\frac{\kappa }{p\hbar^{n}}),\\ & =M(\xi_{1},\xi_{0},\frac{\kappa }{p\hbar^{n+p-1}})\;❃M(\xi_{1},\xi_{0},\frac{\kappa }{p\hbar^{n+p-2}})\;❃\cdots \;❃M(\xi_{1},\xi_{0},\frac{\kappa }{p\hbar^{n}}),\\ & =1\;❃1\;❃\cdots \;\;❃1=1. \end{array}$$

This demonstrates that the sequence *ξ*_*n*_ exhibits Cauchy behavior within Ξ. As, Ξ is the complete fuzzy metric, the sequence {*ξ*_*n*_} is converges to a point *ξ* in Ξ. Since, *ϑ* is continuous, and we assume that *ϑ*(*ξ*) ≠ *ξ*, then there is different closed neighborhoods V and U, such that ξ∈U and ϑ(ξ)∈V and

$$\begin{array}{cc} M(\vartheta \left(\xi_{n}\right),\xi_{n},\kappa ) & =M(\vartheta^{m_{n-1}}\cdot \vartheta \left(\xi_{n-1}\right),\vartheta^{m_{n-1}}\left(\xi_{n}\right),\kappa )\\ & \geq M(\vartheta \left(\xi_{n-1}\right),\xi_{n-1},\kappa )\\ & \vdots \\ & \geq M(\vartheta \left(\xi_{0}\right),\xi_{0},\kappa )\to 1. \end{array}$$

Hence, *M*(*ϑ*(*ξ*_*n*_), *ξ*_*n*_, *κ*) → 1. This means that the lim_*n*→∞_ *ϑ*(*ξ*_*n*_) = lim_*n*→∞_(*ξ*_*n*_), from this we get the fixed point *ξ* of *ϑ* in Ξ.

Next, we establish the uniqueness of the fixed point within Ξ. Take two distinct fixed points *ξ*, *η* in Ξ, such that *ϑ*(*ξ*) = *ξ* and *ϑ*(*η*) = *η*, such that

$$\begin{array}{cc} 1\geq M(\xi ,\eta ,\kappa ) & =M(\vartheta (\xi ),\vartheta (\eta ),\kappa )\\ & \geq M(\xi ,\eta ,\frac{\kappa }{\hbar })\\ & =M(\vartheta \left(\xi \right),\vartheta \left(\eta \right),\frac{\kappa }{\hbar^{2}})\\ & \vdots \\ & \geq M(\vartheta \left(\xi \right),\vartheta \left(\eta \right),\frac{\kappa }{\hbar^{n}}) \end{array}$$

Now, as *n* tends to infinity we get *ξ* = *η*, which complete our result. For, sufficiently large number *n*, we have *n* = *α* ⋅ *n*(*ξ*) + *c*, with *α* > 0, 0 ≤ *c* < *n*(*ξ*), then

$$\begin{array}{cc} M(\vartheta^{n}\left(\xi_{0}\right),\xi ,\kappa ) & =M(\vartheta^{\alpha \cdot n(\xi )+c}\left(\xi_{0}\right),\vartheta^{n(\xi )}\left(\xi \right),\kappa )\\ & =M(\vartheta^{n(\xi )}\cdot \vartheta^{(\alpha -1)n(\xi )+c}\left(\xi_{0}\right),\vartheta^{n(\xi )}\left(\xi \right),\kappa )\\ & \geq M(\vartheta^{(\alpha -1)n(\xi )+c}\left(\xi_{0}\right),\vartheta^{n(\xi )}\left(\xi \right),\frac{\kappa }{\hbar })\\ & \vdots \\ & \geq M(\vartheta^{c}\left(\xi_{0}\right),\xi ,\frac{\kappa }{\hbar^{\alpha }}) \end{array}$$

buy this we get *ϑ*^*n*^(*ξ*_0_) tends to *ξ*, whenever *n* tends to ∞.

Now, we will omit the continuity condition to prove the above result. The subsequent lemma plays a crucial role within this context.

**Lemma 3.2**. *Let a fuzzy metric space* (Ξ, *M*, ❃) *and a self mapping ϑ*: Ξ → Ξ. *If we take* B *a subset of* Ξ, *with* ϑ(B)⊂B. *Then there exist* ξ∈B *and a natural number n*(*ξ*), *such that ϑ*^*n*(*ξ*)^ = *ξ and ϑ satisfy the condition* (5) *(Fuzzy Sehgal Contraction), then ξ represent a fixed point of ϑ in* B *which is unique and ϑ*^*n*^(*η*_0_) → *ξ*, *for each η*_0_ *in* B.

*Proof*. Since, *ξ* is a fixed point of *ϑ*^*n*(*ξ*)^ and *ϑ* is satisfy the Fuzzy Sehgal contraction condition, that gives *ξ* is the unique fuzzy fixed point of *ϑ* in B. Then *ϑ*(*ξ*) = *ϑ*(*ϑ*^*n*(*ξ*)^(*ξ*)) = *ϑ*^*n*(*ξ*)^(*ϑ*(*ξ*)) Now,

$$\begin{array}{cc} M(\vartheta (\xi ),\xi ,\kappa ) & =M(\vartheta \left(\vartheta^{n(\xi )}\left(\xi \right)\right),\vartheta^{n(\xi )}\left(\xi \right),\kappa )\\ & =M(\vartheta^{n(\xi )}\left(\vartheta \left(\xi \right)\right),\vartheta^{n(\xi )}\left(\xi \right),\kappa )\\ & \geq M(\vartheta \left(\xi \right),\xi ,\frac{\kappa }{\hbar })\\ & \vdots \\ & \geq M(\vartheta \left(\xi \right),\xi ,\frac{\kappa }{\hbar^{n}}) \end{array}$$

So, $M(\vartheta \left(\xi \right),\xi ,\frac{\kappa }{\hbar^{n}})\to 1,$ whenever *n* → ∞, for all *κ* ∈ (0, 1), its gives *ϑ*(*ξ*) = *ξ*. The calculation for Uniqueness and *ϑ* → *ξ* are similar to previous theorem.

**Theorem 3.3**. *Let us take a complete fuzzy metric space* (Ξ, *M*, ❃), *and a self mapping ϑ from* Ξ *into itself, and* B *subset of* Ξ, *such that* ϑ(B)⊂B, *and ϑ satisfy the fuzzy sehgal contraction condition and for some* $\xi_{0}\in B$, $cl\{\vartheta^{n}\left(\xi_{0}\right):n\geq 1\}\subset B$. *Then there exist a unique fuzzy fixed point ξ in* B *and ϑ*^*n*^(*η*_0_) → *ξ*, *for each η*_0_ *in* B. *If ϑ satisfy fuzzy sehgal contraction* (5) *in* Ξ, *the result holds in* Ξ.

*Proof*. The convergence of the sequence *ξ*_*n*_ to a point *ξ* in B, similar as in theorem (3.1). So, that *ξ*_*n*_ → *ξ* in B, thus there exist a natural number *n*(*ξ*) such that the *ϑ* satisfy the fuzzy sehgal contraction (5), for each η∈B. So, its gives *ϑ*^*n*(*ξ*)^(*ξ*_*n*_) → *ξ*, by simple calculation we get *ϑ*^*n*(*ξ*)^(*ξ*) = *ξ*, then by Lemma (3.2), *ξ* is unique fuzzy fixed point of *ϑ* in B, and *ϑ*^*n*^(*η*_0_) → *ξ*, for each $\eta_{0}\in B$.

Similarly, if *ϑ* satisfy fuzzy sehgal contraction (5), in Ξ, the result holds in Ξ.

**Example 3.4**. *Let us take* Ξ = [0, 1], *with the fuzzy metric* $M\left(\xi ,\eta ,\kappa \right)=\frac{\kappa }{\kappa +|\xi -\eta |}$, *for all κ* > 0, *with continuous t-norm* “❃”. *Taking a self mapping ϑ*: Ξ → Ξ, *Here we write* $\Xi =\left[0,1\right]=\cup_{ℵ=1}^{\infty }[\frac{1}{2^{ℵ}},\frac{1}{2^{ℵ+1}}]\cup \left\{0\right\}$ *and we define the self mapping for each N* = 1, 2, 3, 4, ⋯,

$$\vartheta :[\frac{1}{2^{ℵ}},\frac{1}{2^{ℵ-1}}]\to [\frac{1}{2^{ℵ+1}},\frac{1}{2^{ℵ}}],$$

*defined by*:

$$\begin{array}{c} \vartheta \left(\xi \right)=\{\begin{array}{cc} \frac{ℵ+2}{ℵ+3}(\xi -\frac{1}{2^{ℵ-1}})+\frac{1}{2^{ℵ}} & \;if\;\;\frac{3ℵ+5}{2^{ℵ+1}\left(ℵ+2\right)}\leq \xi \leq \frac{1}{2^{ℵ-1}}\\ \frac{1}{2^{ℵ+1}}, & \;if\;\;\frac{1}{2^{ℵ}}\leq \xi \leq \frac{3ℵ+5}{2^{ℵ+1}\left(ℵ+2\right)}\\ 0 & \;if\;\;\xi =0. \end{array} \end{array}$$

*We see that ϑ is clearly non-decreasing and continuous function. Here we chose* $\xi \in [\frac{1}{2^{ℵ}},\frac{1}{2^{ℵ-1}}]$ *and η* ∈ Ξ, *we chose* $\eta \in [\frac{1}{2^{ℵ+2}},\frac{1}{2^{ℵ+1}}]$. *If* $\xi \in [\frac{3ℵ+5}{2^{ℵ+1}\left(ℵ+2\right)},\frac{1}{2^{ℵ-1}}]$ *then* $\vartheta \left(\xi \right)=\frac{ℵ+2}{ℵ+3}(\xi -\frac{1}{2^{ℵ-1}})+\frac{1}{2^{ℵ}}$, *and for* $\eta \in [\frac{3(ℵ+2)+5}{2^{ℵ+3}\left(ℵ+4\right)},\frac{1}{2^{ℵ+1}}]$ *then* $\vartheta \left(\eta \right)=\frac{ℵ+4}{ℵ+5}(\eta -\frac{1}{2^{ℵ+1}})+\frac{1}{2^{ℵ+2}}$. *Now, by simple calculation we can calculate*

$$\begin{array}{cc} \left|\vartheta (\xi )-\vartheta (\eta )\right| & =|[\frac{ℵ+2}{ℵ+3}(\xi -\frac{1}{2^{ℵ-1}})+\frac{1}{2^{ℵ}}]-[\frac{ℵ+4}{ℵ+5}(\eta -\frac{1}{2^{ℵ+1}})+\frac{1}{2^{ℵ+2}}]|\\ & =|\frac{ℵ+2}{ℵ+3}\left(\xi \right)-\frac{ℵ+4}{ℵ+5}\left(\eta \right)-\frac{1}{2^{ℵ-1}}[\frac{6{ℵ}^{2}+36ℵ+22}{16(ℵ+3)(ℵ+5)}]|\\ & \leq |\frac{ℵ+2}{ℵ+3}\left(\xi \right)-\frac{ℵ+4}{ℵ+5}\left(\eta \right)|\\ & \leq \frac{ℵ+4}{ℵ+5}\;|(\xi )-(\eta )| \end{array}$$

*By this we get*,

$$M(\vartheta \left(\xi \right),\vartheta \left(\eta \right),\frac{ℵ+4}{ℵ+5}\left(\kappa \right))\geq M\left(\xi ,\eta ,\kappa \right).$$

*But if we chose* $\xi \in [\frac{1}{2^{ℵ}},\frac{3ℵ+5}{2^{ℵ+1}\left(ℵ+2\right)}]$ *then* $\vartheta \left(\xi \right)=\frac{1}{2^{ℵ+1}},$ *and for* $\eta \in [\frac{1}{2^{ℵ+2}},\frac{3(ℵ+2)+5}{2^{ℵ+3}\left(N+4\right)}]$ *then* $\vartheta \left(\eta \right)=\frac{1}{2^{ℵ+3}}$ , *from these we get*

$$\begin{array}{cc} \left|\vartheta (\xi )-\vartheta (\eta )\right| & =|\frac{1}{2^{ℵ+1}}-\frac{1}{2^{ℵ+3}}|\\ & =\frac{3}{2^{ℵ+3}} \end{array}$$

*and we see that*

$$\left|\xi -\eta \right|=|\frac{1}{2^{ℵ}}-\frac{1}{2^{ℵ+1}}|=\frac{1}{2^{ℵ+1}}$$

*So, its clearly*

$$\frac{3}{2^{ℵ+3}}\geq \hbar (\frac{1}{2^{ℵ+2}}),\;\;\;for\;\;\hbar =\frac{1}{2},$$

*that’s gives that*

M(ϑ(ξ),ϑ(η),ℏκ)≤M(ξ,η,κ)

*So, ϑ is not contraction. Now*, $\vartheta^{2}\left(\xi \right)=\frac{1}{2^{ℵ+2}}$ *and ϑ*^2^(2^ℵ+4^), *then we can find*

$$|\vartheta^{2}\left(\xi \right)-\vartheta^{2}\left(\eta \right)|=\frac{3}{2^{ℵ+4}}$$

*so, that*

$$M\left(\vartheta \left(\xi \right),\vartheta \left(\eta \right),\hbar \kappa \right)\geq M\left(\xi ,\eta ,\kappa \right),\;\;\;with\;\;\hbar =\frac{1}{2}.$$

*So, ϑ*^2^ *is a fuzzy contraction. Therefore, we can say ϑ is Fuzzy Sehgal Contraction and there is a unique fixed point of ϑ is* 0.

A detailed analysis can be seen in the table the following Table 1, which shows that our results’ convergence rate is significantly faster than that of the Picard operator’s. We have selected an initial guess, *x*_0_ = 1 and chose *n* = 1, and agreed to attain accuracy up to six decimal places.

**Table 1:** Comparison between Picard and Sehgal.

S. No.	Sequence *x*_*n*_	Picard Operator	Sehgal Operator
00	*x*_0_	1	1
01	*x*_1_	0.5	0.0625
02	*x*_2_	0.25	0.000488
03	*x*_3_	0.125	0.000000
04	*x*_4_	0.0625	0.000000
05	*x*_5_	0.03125	0.000000
06	*x*_6_	0.015625	0.000000
07	*x*_7_	0.007813	0.000000
08	*x*_8_	0.003906	0.000000
09	*x*_9_	0.001953	0.000000
10	*x*_10_	0.000977	0.000000
11	*x*_11_	0.000488	0.000000
12	*x*_12_	0.000244	0.000000
13	*x*_13_	0.000122	0.000000
14	*x*_14_	0.000061	0.000000
15	*x*_15_	0.000031	0.000000
16	*x*_16_	0.000015	0.000000
17	*x*_17_	0.000007	0.000000
18	*x*_18_	0.000004	0.000000
19	*x*_19_	0.000002	0.000000
20	*x*_20_	0.000001	0.000000
21	*x*_21_	0.000000	0.000000

**Fig 1:**
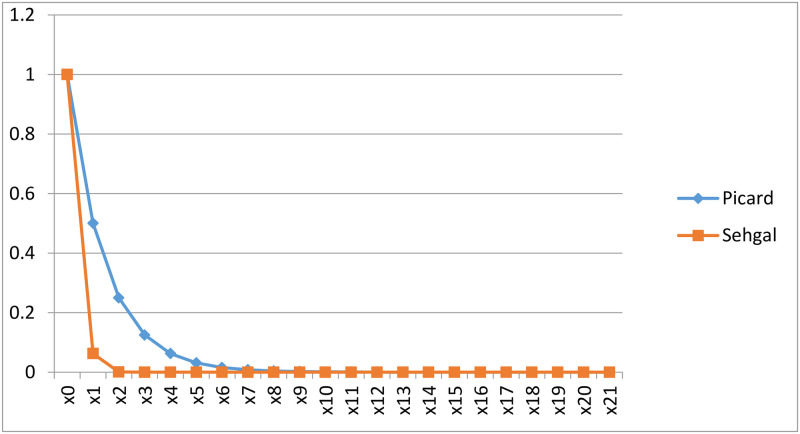
Comparison between Picard and Sehgal.

We present a graphically representation of convergence comparison of the sequences produced by Picard and Sehgal in Fig 1. as follows:

## 4 Application

When the metric space under investigation is a function space, the applications of fixed point theorems become especially intriguing. In this context, we use Theorem 3.1 to investigate the existence and uniqueness of the Volterra integral equation.

If *Υ* is a continuous function defined on the space [*a*, *b*] × [*a*, *b*], and *ϕ* is continuous on the interval [*a*, *b*], then the Volterra equation is defined as:

$$\begin{array}{c} f\left(\xi \right)=\phi \left(\xi \right)+\Lambda \int_{a}^{\xi }ϒ\left(\xi ,\eta \right)f\left(\eta \right)d\eta \end{array}$$ (6)

where Λ is the parameter.

**Theorem 4.1**. *For each* Λ∈R, *the Volterra equation* (6) *has a unique solution* f *that is continuous on* [*a*, *b*].

*Proof*. Let Ξ = *C*[*a*, *b*] the set of all continuous real-valued functions defined on [*a*, *b*] with the metric $M\left(\xi ,\eta ,\kappa \right)=\frac{\kappa }{\kappa +|\xi +\eta |}$. Since *Υ* is continuous, there exist a constant *ζ* > 0 such that |*Υ*(*ξ*, *η*)| ≤ *ζ* for all *ξ*, *η* ∈ [*a*, *b*]. Define a transformation $\vartheta :f\to \vartheta^{\left(n(f)\right)}\left(f\right)$ on Ξ by

$$\begin{array}{c} \vartheta^{\left(n(f)\right)}\left(f\left(\xi \right)\right)=\phi \left(\xi \right)+\Lambda \int_{a}^{\xi }ϒ\left(\xi ,\eta \right)f\left(\eta \right)d\eta \end{array}$$ (7)

for all *ξ*, *η* ∈ Ξ, we have

$$\begin{array}{cc} \left|\vartheta^{n(f)}\left(f\left(\xi \right)\right)-\vartheta^{n(f)}\left(g\left(\xi \right)\right)\right| & =|\Lambda \int_{a}^{\xi }ϒ\left(\xi ,\eta \right)\left|f\left(\eta \right)-g\left(\eta \right)\right|d\eta |,\\ & \leq \left|\Lambda ||\upsilon \left(\xi -\eta \right)||f\left(\xi \right)-g\left(\eta \right)\right|\int_{a}^{\xi }d\eta ,\\ & \leq |\Lambda |\zeta (\xi -a)|f(\eta )-g(\eta )| \end{array}$$

we get

$$\kappa +|\vartheta^{n(f)}\left(f\left(\xi \right)\right)-\vartheta^{n(f)}\left(g\left(\xi \right)\right)|\leq \kappa +|\Lambda |\zeta \left(\xi -a\right)|f\left(\eta \right)-g\left(\eta \right)|$$

From this we get

$$\frac{\kappa }{\kappa +|\vartheta^{n(f)}\left(f\left(\xi \right)\right)-\vartheta^{n(f)}\left(g\left(\xi \right)\right)|}\geq \frac{\kappa }{\kappa +|\Lambda |\zeta (\xi -a)|f(\eta )-g(\eta )|}$$

this implies

$$M\left(\vartheta^{n(f)}f\left(\xi \right),\vartheta^{n(f)}\left(g\left(\xi \right)\right),\kappa \right)\geq M(f,g,\frac{\kappa }{\left|\Lambda |\zeta (\xi -a\right)})$$

Which shows that Volterra integral equation has a unique solution.

## 5 Conclusion

In this work we have introduced the Sehgal fuzzy contraction and established some fixed point results in fuzzy complete metric space. We have proved results using continuity condition and also omitting the continuity condition. We have constructed a non-trivial example to validate our result. Additionally, we carefully analyzed the sequence in our findings using both numerical and graphical methods in order to compare its convergence with the Picard operator. A detailed graphical comparison has been conducted. The outcomes clearly show that our method has a higher rate of convergence than the Picard method.Finally, we applied our methodology to establish the existence and uniqueness of solutions for the Volterra integral equation, thereby enhancing the robustness of our findings.

### 5.1 Future direction

In the future, we plan to enhance the efficiency of contractions further. Additionally, we aim to validate these results across various spaces and for different types of mappings.
